# Joint Meeting of the American Dental Association and the Southern Dental Association

**Published:** 1888-10

**Authors:** William Conrad

**Affiliations:** St. Louis, Mo.


ARTICLE III.

### JOINT MEETING OF THE AMERICAN DENTAL ASSOCIATION AND THE SOUTHERN DENTAL ASSOCIATION.

REPORTED BY WILLIAM CONRAD, D. D. S., ST. LOUIS, MO.

The joint meeting of the American Dental Association and the Southern Dental Association was held at Louisville, Ky., August 28th and 31st., inclusive. Frank Abbott, M. D., New York City, President of the American Dental Association, and B. H. Catching, D. D. S., Atlanta, Ga., President of the Southern Dental Association, both being present and fulfilling the duties laid down by the programme with credit to themselves and profit to the meeting.

The rule governing the joint meeting was as follows: "As the joint session is only for scientific and social purposes, nothing but professional subjects will be discussed or acted upon."

The Female High School building was well adapted for the work of the Associations, and the local committee deserve special mention for the efficient manner in which they attended to the arduous duties imposed upon them.

The visiting dentists received every attention from the hotels, the people of Louisville, and the railroads leading into the city.

The attendance of the American Association membership was limited as compared with the Southern Association. The meeting was conspicous for the large numbers of Western and Southern dentists, and the lack of Eastern "Wheel Horses." It was easy to see from where the profession of the future is to draw its active workers.

The entire absence of Clinics of any kind was a constant source of regret. There had been ample provisions made for practical work, and its loss will be felt by many who are not so "scientifically high-toned" that they are above making better dentists of themselves.

If it had not been for the enterprise of the dental dealers and some few individual practitioners, the meeting would have been devoid of any actual benefit so far as practical work is concerned. The displays were excellent, and the desire to sell goods in order to pay "heavy expenses," proved the spirit of competition to be a source of gain for the every-day dentist. The S. S. White Dental Manufacturing Co., with its efficient Tom Long and Dr. Selby of "The Dental Chewing Gum" fame, as representatives, assisted by the veteran editor, Dr. White, of the *Cosmos*, proved too much for the lesser lights in the Dealers Association. The White Company secured accommodations at the entrance and managed to capture many before they could pass the fortifications. They deserved success, and worked hard for it. The Sibley Dental Co., represented by young Dr. Sibley; the American Dental Manufacturing Co., with its president, Hanway; the Welch Dental Manufacturing Co.; the Florence Manufacturing Co.; C. Ash & Co., of London and New York, with a fine line of English goods; Wilmington Dental Co., represented by Brewster, formerly proprietor of the Kansas City Dental Depot, etc.

The room devoted to electrical appliances was one of the main attractions, and proved the interest the profession is taking in some form of motor. Dentists are getting tired of doing so much manual labor, and electricity is being put forward as a candidate for dental favor. The electric battery has almost been admitted a dead failure in the hands of most practicing dentists. They will not, or can not give it the time to make it a satisfactory source of motive power. The direct wire, or the storage battery, seems to be the form most to be relied upon for constant work. Dr. Knapp of New Orleans, had his complete office outfit; it was pretty, but looked dangerous. It would take an electrical engineer to pull the strings and work the wires, and in our Western country it would scare away most ordinary patients; still if we could overcome this objection, it might cause some of them to extend our reputation by talking. Dr. Kells, of New Orleans, had on exhibition his office fixtures; they appeared to be very convenient and effective. The Detroit and Belding Motors were ably handled by their respective representatives. The Wooley Motor seemed to meet with general favor.

The papers were prepared for the "Transactions," and will be more interesting when considered deliberately. They were presented in groups, and the points of one paper would be lost while listening to another, consequently the discussion was most always off the subject

Members present who were accustomed to attend society meetings, said they had heard the same things repeated for the past ten years.

Dr. Frank Abbott's address as President of the American Association, on "Dental Education," was the first paper of the meeting.

Dr. B. H. Catching's President's address, "Is the Average Dentist of to-day a Specialist in Medicine?" came next in order.

Dr. Abbott's paper, although of much interest and value, was not discussed, while that of Dr. Catching brought all the College Professors to their feet, it seemed as if to defend their pocket-books.

The Commercial Club, of Louisville, invited the members to an excnrsion, but the rain prevented its taking place. The club extended to the members the freedom of their club-rooms. The thanks of the Association was extended for these invitations.

Dr. W. S. How, of the S. S. White Dental Manufacturing Co., had a paper on "Dental Repair by the Employment of Inlays."

Dr. John J. R. Patrick, Belleville, Ill., presented an interesting paper on "Artificial Crowns."

Dr. Chas. E. Kells, Jr., of New Orleans, La., demonstrated the effect of heat and cold upon the various materials used for capping pulps, making use of an electric apparatus for this purpose. Gutta-percha proved to be the best non-conductor under his tests.

Dr. T. L. Gilmer, Quincy, Ill., read a paper on "A New and Cheap Way to Make Crowns."

Dr. I. P. Wilson, Burlington, Iowa, paper, "The Apical Portion of Cementum Physiologically and Pathologically Considered." This was very highly complimented. He cautioned his hearers against the use of arsenic in dental surgery, and especially against its use twice in the same operation.

Dr. Frank Abbott, gave the Association the result of his investigations in a paper on "Odontoblasts in their Relation to Developing Dentine." Drs. Abbott and Sudduth entered into a personal discussion upon the relative merits of American and European microscopical work, as shown in their microscopical slides.

Many members visited Mammoth Cave, absenting themselves from the meeting, and setting a bad example to others who might attend.

Dr. A. W. Harlan read a paper by Dr. Smith, of Paris, France, on the "Use of Cocaine, Administered Hypodermically."

Dr. L. G. Noel, Nashville, Tenn., made a report on "Dental Materia Medica."

Dr. Louis Ottofy, Chicago, Ill., presented a report on "Incipient Decay," which showed great industry on the part of the essayist.

Dr. A. W. Harlan, Chicago, Ill., had a paper on "New Remedies."

Dr. H. A. Smith, Cincinnati, Ohio, gave the Association one of the best papers of the meeting, on "Dental Implantation," together with a tabulated statement, the result of his investigation, which showed that about seven per cent. of the cases reported were failures.

Dr. J. D. Patterson, Kansas City, Mo., paper, "Pyorrhœa Alveolaris." Dr. Patterson has made a special study of this subject for a number of pears, and has presented several valuable papers, the present one being a continuation of the subject. His theory is unique, but has as much foundation in fact as any presented to the profession; it being the result of many years investigation.

Dr. W. W. Allport was allowed the privilege of the floor to make an explanation of a matter which concerned him personally.

Dr. T. W. Brophy, Chicago, Ill., made a report on "Anatomy, Pathology and Surgery."

Dr. John S. Marshall, Chicago, Ill., presented a "Report on Some Cases in Hospital Practice."

Dr. J. Rolla Knapp, New Orleans, La., read a paper on "Cocaine." Its use was condemned by the Committee, owing to its dangerousness, sustaining its position by quotations from English dentists.

Dr. Genese, Baltimore, Md., a paper on "Rubber."

Dr. John S. Marshall, of Chicago, offered the following interesting resolution:

*Whereas*, It is the sense and belief of the American Dental and Southern Dental Associations in joint meeting assembled, that the tariff on imported dental and surgical goods works a hardship upon the profession and the public. Therefore, be it

*Resolved*, That we memorialize Congress to abolish all duties upon imported dental and surgical instruments, apparatus and supplies.

*Resolved*, That the secretaries of the Associations be instructed to forward these resolutions to the proper authorities in Congress, and that each member of the Association be requested to use his influence with the Congressman from his district to place the above-mentioned goods upon the free list.

The resolutions were greeted with applause, and Dr. J. C. Story of Texas, moved that they be adopted as read. The motion was carried by an almost unanimous vote.

Dr. J. Taft read a paper by Dr. J. Holly Smith, Baltimore, Md., on "Dental Education."

Dr. Louis Ottofy, Dr. Wm. H. Atkinson and Dr. L. C. Ingersoll read papers on the same subject.

Dr. Geo. J. Friedricks, New Orleans, La., read a paper on "Dental Hygiene." It was unfortunate that this paper came last on the list, as it was well worthy the consideration of a full meeting. The writer proved himself one of the bright lights of dentistry in the South.

OFFICERS OF THE AMERICAN AND SOUTHERN DENTAL ASSOCIATIONS FOR 1888.

The American Dental Association elected the following officers for 1888-9:

President, Dr. C. R. Butler, Cleveland, Ohio; First Vice-President, Dr. A. W. Harlan, Chicago, Ill.; Second Vice-President, Dr. S. A. White, Savannah, Ga.; Corresponding Secretary, Dr. F. A. Levy, Orange, N. J.; Recording Secretary, Dr. Geo. H. Cushing, Chicago, Ill.; Treasurer, Dr. A. H. Fuller, St. Louis, Mo.; Executive Committee, Drs. Darby, McElhany, Abbott and Crouse.

Next place of meeting, Saratoga, N. Y., August, 1889.

The Southern Dental Association elected the following officers for the ensuing year:

President Dr. J. Y. Crawford, Nashville, Tenn.; First Vice-President, Dr. John C. Story, Dallas, Texas; Second Vice-President, Dr. Wm. N. Morrison, St. Louis, Mo.; Third Vice-President; Dr. John S. Thompson, Atlanta, Ga.; Recording Secretary, Dr. M. C. Marshall, Little Rock, Ark.; Corresponding Secretary, Dr. D. R. Stubblefield, Nashville, Tenn.; Treasurer, Dr. H. A. Lowrance, Athens, Ga.

Next place of meeting, Galveston, Texas, third Tuesday in August, 1889.—*The Archives of Dentistry.*

